# Stage B Heart Failure Is Ubiquitous in Emergency Patients with Asymptomatic Hypertension

**DOI:** 10.5811/westjem.17990

**Published:** 2024-02-09

**Authors:** Kimberly Souffront, Bret P. Nelson, Megan Lukas, Hans Reyes Garay, Lauren Gordon, Thalia Matos, Isabella Hanesworth, Rebecca Mantel, Claire Shubeck, Cassidy Bernstein, George T. Loo, Lynne D. Richardson

**Affiliations:** * Icahn School of Medicine at Mount Sinai, Department of Emergency Medicine, New York; † Center for Nursing Research and Innovation, Mount Sinai Health System, New York; ‡ Ichan School of Medicine at Mount Sinai, Institute for Health Equity Research, New York; § Mount Sinai Morningside, Department of Cardiology, New York; ∥ Icahn School of Medicine at Mount Sinai, Department of Population Health Science and Policy, New York; ¶ Icahn School of Medicine at Mount Sinai, Department of Artificial Intelligence and Human Health, New York

## Abstract

**Introduction:**

Hypertension is the leading risk factor for morbidity and mortality throughout the world and is pervasive in United States emergency departments (ED). This study documents the point prevalence of subclinical heart disease in emergency patients with asymptomatic hypertension.

**Method:**

This was a prospective observational study of ED patients with asymptomatic hypertension conducted at two urban academic EDs that belong to an eight-hospital healthcare organization in New York. Adult (≥18 years of age) English- or Spanish-speaking patients who had an initial blood pressure (BP) ≥160/100 millimeters of mercury (mmHg) and second BP ≥140/90 mm Hg, and pending discharge, were invited to participate in the study. We excluded patients with congestive heart failure, renal insufficiency, and atrial fibrillation, or who were pregnant, a prisoner, cognitively unable to provide informed consent, or experiencing symptoms of hypertension. We assessed echocardiographic evidence of subclinical heart disease (left ventricular hypertrophy, and diastolic and systolic dysfunction).

**Results:**

A total of 53 patients were included in the study; a majority were young (mean 49.5 years old, [SD 14–52]), self-identified as Black or Other (n = 39; 73.5%), and female (n = 30; 56.6%). Mean initial blood pressure was 172/100 mm Hg, and 24 patients (45.3%) self-reported a history of hypertension. Fifty patients completed an echocardiogram. All (100%) had evidence of subclinical heart disease, with 41 (77.4%) displaying left ventricular hypertrophy and 31 (58.5%) diastolic dysfunction. There was a significant relationship between diastolic dysfunction and female gender [x^2^ (1, n = 53) = 3.98; *P* = 0.046]; Black or other race [x^2^ (3, n = 53) = 9.138; *P* = 0.03] and Hispanic or other ethnicity [x^2^ (2, n = 53) = 8.03; *P* = 0.02]. Less than one third of patients demonstrated systolic dysfunction on echocardiogram, and this was more likely to occur in patients with diabetes mellitus [x^2^ (1, n = 51) = 4.84; *P* = 0.02].

**Conclusion:**

There is a high probability that Black, Hispanic, and female patients with asymptomatic hypertension are on the continuum for developing overt heart failure. Emergency clinicians should provide individualized care that considers their unique health needs, cultural backgrounds, and social determinants of health.

## INTRODUCTION

Hypertension is the leading risk factor for morbidity and mortality throughout the world and is pervasive in United States emergency departments (ED).^1^ ^,^ ^2^ It is a common misconception that high blood pressure found during the ED visit is related to pain or anxiety; however, studies show that elevated blood pressure in the ED reliably identifies hypertension in an overwhelming majority of patients^3^ and is an independent risk factor for having a major cardiovascular event after discharge.^4^

The clinical policy recommended by the American College of Emergency Physicians (ACEP) for patients who have persistent asymptomatic hypertension in the ED *without* signs and symptoms of acute target organ injury includes prompt referral to primary care.^5^ However, this clinical policy is outdated and inconsistently adhered to.^6^ Furthermore, of the patients who are referred, less than half adhere to recommendations regarding follow-up, regardless of their insurance status or access to routine medical care.^7^ Automating recommendations for follow-up using the electronic health record is one way to ensure adherence to the ACEP policy;^8^ however, this is not useful or meaningful to patients when it is not followed by any clinician-to-patient communication about the importance of follow-up. Besides, this policy does not consider the emerging evidence that an overwhelming majority of emergency patients, particularly Blacks, with asymptomatic hypertension show evidence of subclinical heart disease,^9^ ^,^ ^10^ with upper limits reaching more than 90.7% in one sentinel study by Levy et al.^9^

This study documents the point prevalence of subclinical heart disease in the first 50 ED patients enrolled in an ongoing study. This was our first step to explore unique ways for improving healthcare delivery for this high-risk patient population in our ED.

## METHODS

### Study Design, Sample, and Setting

This was a prospective observational study of emergency patients with asymptomatic hypertension. Data collection began after receiving institutional review board approval (#18-00197). The study was conducted at two urban academic EDs that provide care to a diverse patient population, serving the local communities of Harlem, New York City. Harlem has a concentrated burden of hypertension and is among the poorest neighborhoods in New York City. More than two-thirds of the residents are a racial or ethnic minority.^11^

Data collection began in 2018 at one ED site and stopped twice due to 1) lack of funding to continue participant recruitment, and 2) a pause in research activities at the recruiting institution in response to the COVID-19 pandemic. In late 2021, an additional site was added to boost patient recruitment. Annually, there are about 100,000 patient visits in each ED, and there is a state-of-the-art electronic information system to facilitate data collection across sites. The estimated prevalence of patients who visit the ED at each site who have uncontrolled asymptomatic hypertension is 48%–50%.^12^

Adult (≥18 years of age) English- or Spanish-speaking patients, who had an initial blood pressure (BP) ≥160/100 millimeters of mercury (mm Hg) and second BP ≥140/90 mm Hg and were pending discharge, were invited to participate in the study. We excluded patients with congestive heart failure, renal insufficiency, and atrial fibrillation, or who were pregnant, a prisoner, cognitively unable to provide informed consent, or experiencing symptoms of hypertension, such as chest pain, paresthesia, or shortness of breath.

#### Protocol Change

Initially we also excluded patients if they had taken blood pressure medication within the prior three months; however, beginning at study participant number 44, we modified our protocol to include all patients regardless of whether they had taken medication within the previous three months. We revised the study protocol to be more inclusive, specifically to avoid excluding patients who might have untreated conditions and to ensure that all individuals, including those potentially in need of intervention, could be considered in our research. This change in protocol allowed for a more comprehensive assessment of the study population and also assisted with our recruitment.

### Data Collection Procedure

Approximately three days/week from 8 am to 4 pm, a research coordinator enrolled and obtained informed consent electronically from patients who met inclusion criteria. Study data were collected and managed using REDCap (Research Electronic Data Capture)^13^ ^,^ ^14^ electronic data capture tools hosted at our institution.^1^ ^,^ ^2^ REDCap is a secure, web-based software platform designed to support data capture for research studies, providing 1) an intuitive interface for validated data capture; 2) audit trails for tracking data manipulation and export procedures; 3) automated export procedures for seamless data downloads to common statistical packages; and 4) procedures for data integration and interoperability with external sources.^13^ ^,^ ^14^

Each morning of patient recruitment, the research coordinator used Microsoft Teams (Microsoft Corp, Redmond, WA) to inform the sonographer that recruitment had begun.^15^ Before approaching any patient in the ED, the research coordinator used the Epic chat feature (Epic Systems Corp, Verona, WI) to inform the ED attending physician that his/her patient met inclusion for the study and would be approached.^15^ If an electrocardiogram (ECG) was not conducted as part of usual care, the ECG was conducted by the research coordinator. The bedside echocardiogram was conducted by one member of the study ultrasound team, which included one ED sonographer, four emergency physicians, and one cardiologist, each trained on the study protocol. Each echocardiogram took approximately 10 minutes to complete at bedside.

### Measures

#### Demographic Measures

Study measures were obtained by electronic data extraction and included the patient’s first and second systolic and diastolic BP level since arrival to the ED, age, gender, race/ethnicity, past medical history, smoking history, and access to primary care. All data were double entered into the REDCap database by two members of the research team to assure accuracy of chart review and data entry. Our protocol was set such that any differences were reconciled by the principal investigator (KS); however, no differences were identified.

#### Echocardiogram

Three endpoints were obtained and modeled after the American College of Cardiology, American Heart Association (ACC/AHA), Heart Failure Society of America, and Levy et al.^9^ ^,^ ^20^:

1. *Left ventricular hypertrophy (LVH)*. Defined as present if left ventricular septal thickness was ≥ 1.1 centimeter (cm) or absent if left ventricular septal thickness was <1.1 cm. (Left ventricular free wall thickness was measured in the parasternal long view at end diastole.)
2. *Systolic dysfunction.* Ejection fraction noted as normal (≥55%) or abnormal (<55%).
3. *Diastolic function.* Evaluated by estimating left atrial pressure using the E/e’ ratio. Diastolic dysfunction was present if E/e’(septal) was ≥15, if E/e’(lateral) was ≥12, if septal e’ was <8 cm/sec, or if lateral e’ was <10 cm/sec.^3^ Grade of diastolic dysfunction was not determined for this initial analysis.

#### Electrocardiogram

The study diagnosed LVH with ECG findings that satisfied the Cornell voltage criteria (when the sum of the R wave in lead aVL and the S wave in lead V3 is >20 mm in women and 23 mm in men). Data obtained from the ECG included heart rate, PR interval, and the QRS interval number.

### Data Analysis Plan

We exported data from REDCap to SAS analytic software version 9.4 (SAS Inc, Cary, NC) for data analysis. Demographic variables are presented as percentages (%) or means (*M*) and standard deviations. Evidence of subclinical heart disease was dichotomized as abnormal/normal and presented as percentages. We conducted bivariate analyses to test for significant relationships between independent variables (age, gender, race/ethnicity, past medical history, body mass index, smoking history, and access to primary care) and subclinical heart disease.

## RESULTS

### Characteristics of Sample

A total of 53 patients were examined. (Two patients did not complete an echocardiogram and one patient did not complete an echocardiogram and ECG due to time constraints.) The majority of the sample were young (mean 49.5 years old, [SD 14–52]), self-identified as Black or Other (n = 39; 73.5%), and female (n = 30; 56.6%). 31 were Hispanic race (58.5%) and 30 female (56.6%). Mean initial BP was 172/100 mm Hg, and 24 patients (45.3%) self-reported a history of hypertension; 36 (68%) had taken their antihypertensive medication within the prior three months. Mean body mass index was 31.9, which correlates to Obesity Class I or moderately obese.^17^ Fifteen patients (28.3%) were current smokers, and 12 (28.3%)] had a history of diabetes mellitus. Thirty-one (58.5%) reported they had a primary care physician (Table 1).

**Table 1.** Demographic characteristics and subclinical heart disease.

Measure	Variable	Statistic N = 53 (%)
Independent variables – demographic characteristics	Age, mean (SD)	49.47 (14.5)
	Gender, n (%)	
	Female	30 (56.6)
	Male	23 (43.4)
	Race, n (%)	
	White	5 (9.4)
	Black or other	48 (90.6)
	Ethnicity, n (%)	
	Non-Hispanic	27 (49.1)
	Hispanic or other	31 (58.5)
	Blood pressure, mean mm Hg	
	1^st^ systolic blood pressure	172.13 (15.1)
	1^st^ diastolic blood pressure	100.74 (15.0)
	2^nd^ systolic blood pressure	162.27 (17.5)
	2^nd^ diastolic blood pressure	95.37 (16.5)
	Hypertension history, yes	24 (45.3%)
	Took blood pressure medicine within 3 months	36 (68.0%)
	Smoker, yes	15 (28.3%)
	Diabetes mellitus history, yes	12 (22.6%)
	Body mass index, mean (SD)	31.93 (8.9)
	Primary care physician, yes	31 (58.5)
Dependent variable – subclinical heart disease	Subclinical heart disease, yes	50 (100)
	Diastolic dysfunction, yes	31 (58.5)
	Left ventricular hypertrophy, yes	41 (77.4)
	Systolic dysfunction, yes	10 (19.6)

### Echocardiogram

Of the 50 patients who completed an entire echocardiogram, 100% had evidence of subclinical heart disease; 41 (77.36%) showed evidence of LVH and 31 (58.49%) diastolic dysfunction. We performed a chi-square test of independence to assess the relationship between subclinical heart disease and our independent variables. There was a significant relationship between diastolic dysfunction and female gender [x^2^ (1, n = 53) = 3.98; *P* = 0.046]; Black or other race [x^2^ (3, n = 53) = 9.138; *P* = 0.03] and Hispanic [x^2^ (2, n = 53) = 8.03; *P* = 0.02]. Less than one-third of patients demonstrated systolic dysfunction on echocardiogram, and this was more likely to occur in patients with diabetes mellitus [x^2^ (1, n = 51) = 4.84; *P* = 0.02]. There were no other differences between our independent variables and our main outcome, subclinical heart disease.

One participant was missing an ECG. Of 49 participants, five demonstrated evidence of LVH.

## DISCUSSION

We found that subclinical heart disease is ubiquitous in ED patients with asymptomatic hypertension. Our findings are consistent with those of Levy et al (2012), who were the first to document the alarming prevalence of structural heart changes in ED patients with asymptomatic hypertension, particularly among Blacks who sought care in the ED of an inner city.^9^ Our study adds to the existing literature that Black and Hispanic patients who have asymptomatic hypertension diagnosed in the ED have a high probability of having myocardial target organ damage, that is consistent with a diagnosis of Stage B heart failure according to the ACC/AHA.^18^

Congestive heart failure is a progressive disorder (Stages A, B, C, and D) that often begins with left ventricular systolic dysfunction and results in symptoms from fluid overload and poor end-organ perfusion.^18^ Stage B heart failure encompasses patients who are asymptomatic and have evidence of structural heart abnormalities, such as LVH, systolic or diastolic dysfunction.^18^ Asymptomatic left ventricular systolic dysfunction has an estimated prevalence of 3–6% in the community,^19^ which is significantly less than our study findings of 19%. Prendergast et al^10^ (2015) found the point prevalence of subclinical heart disease in emergency patients with asymptomatic hypertension to be nearly 40%, also significantly less than our findings of 100%. To our knowledge, no other study exists that documents the point prevalence of subclinical heart disease in ED patients with asymptomatic hypertension. Both Levy et al (2012) and Prendergast et al (2015) conducted their studies in inner-city Detroit and inner-city Chicago, respectively—settings similar to ours.^9^ ^,^ ^10^

In randomized trials, individuals with asymptomatic left ventricular systolic dysfunction have high rates of incident heart failure and death, increased cardiovascular mortality^19^ ^–^ ^21^; all-cause mortality^20^; and nonfatal cardiovascular events, such as myocardial infarction and stroke.^20^ ^,^ ^22^ While the relative contribution of each condition (systolic and diastolic dysfunction, or LVH) to overt congestive heart failure is unknown, from the lens of emergency medicine and population health, identifying patients with asymptomatic hypertension is of utmost importance, regardless.

Our study demonstrates that ED patients with asymptomatic hypertension are on the continuum to overt heart failure, which has serious consequences. A considerable number of missed opportunities occur, which contribute to delays in diagnosis, suboptimal treatment, increased morbidity and mortality and, above all, an exacerbation of disparities related to hypertension among Blacks. Studies suggest referral rates are 5%.^12^ Despite the many knowledge, attitudinal, and organizational barriers that exist,^6^ ^,^ ^23^ ^,^ ^24^ the identification and management of asymptomatic hypertension is critical to prevent the progression of the disease and its complications.

## LIMITATONS

The results of our study must be interpreted considering its limitations, which include its small sample size and study design. We analyzed only the first 50 patients of an ongoing study, which limits the ability to generalize to larger populations. Additionally, our prospective observational study design limits the ability to draw conclusions about the progression of congestive heart failure or other cardiovascular events over time. Lastly, we did not evaluate subclinical heart disease in normotensive patients. Nonetheless, this study has strengths in that it documents the significant burden of Stage B heart failure in the emergency population with asymptomatic hypertension.

## IMPLICATIONS

Emergency clinicians should recognize the prevalence of asymptomatic hypertension in Black patients and provide individualized care that considers their unique health needs, cultural backgrounds, and social determinants of health. This includes providing adequate follow-up care and support to prevent hypertension-related complications and improve overall health outcomes.

## CONCLUSION

In this study we documented the point prevalence of subclinical heart disease in the first 50 ED patients who were enrolled in an ongoing study, as our first step to explore unique ways for improving healthcare delivery for this high-risk patient population in our emergency department. While subclinical heart disease was ubiquitous, we conclude that female, Black, and Hispanic patients who have asymptomatic hypertension diagnosed in the ED have a high probability of already being on the continuum for developing overt heart failure.
